# Drugs and Their Uses

**Published:** 1894-03-10

**Authors:** 


### Drugs and Their Uses.

Next to his "Practice of Physic," the working practioner requires most of all an intelligent and informing book on Materia Medica. The drugs which the doctor uses are his weapons of war. Any tyro can tell us that good weapons, skilfully handled, generally make the scale dip on the side of victory. On the other hand, rude weapons unskilfully used most certainly court defeat in a serious encounter. There are few things of which less is scientifically known than of the action of drugs in general. But it does not require the mind of a philosopher to perceive that few things can be more desirable than that the practising doctor should know all about his drugs in all their moods and tenses. It is in some respects fortunate that the average practitioner confines his attention in practice to a strictly limited number of remedies; and these, by constantly prescribing he becomes so entirely familiar with that it is hardly possible for him to go wrong. On the other hand, this limitation in the range of his resources is cramping to his own mind, and by no means always the most satisfactory thing in the world for his patient. It is in every respect desirable therefore that progress in the scientific knowledge and use of drugs should be made as easy as possible to practitioners old and young.

Dr. Hale White, in his "Materia Medica" just published, has, we are convinced, done his "level best," as the Americans say, to place the fruits of a competent experience and an amount of knowledge quite uncommon at the service of every practitioner who can use a little diligence in study and a little common sense in practice. The book is by no means a routine text book, and should not be so received by the profession. It is a book into which the author has put his strength, and it will convince every honest reader that that "strength" is much above the average.

We are conscious as we study the little volume—little, that is, by comparison with the interminable tomes of past days—that Dr. White has done in his study what we all have to do at the bedside, namely, set his patient over against the drugs to be employed in his treatment, and asked himself what the latter are going to do for the former. "Here is the patient," he seems to say, "let us consider him well. There are the drugs, let us also consider them well; and let us by some means try to make an advantageous, pleasant, and permanently successful union between the two." The two problems are not easy; the problem of the physic is very difficult; the problem of the patient is more difficult still. Dr. White has not shirked either the one or the other. He has done his clearest, his simplest, his briefest and his best to render his brother practitioners a real service in physic's art.

The work is most business-like. "Let us begin at the chemist's shop," it seems to say, "or better still, in the laboratory of the scientific compounder. Let us look at his materials and his methods, at the scientific tools of his trade, and at his various, interesting, and often admirable results. Let us acquire some actual knowledge of modern medical materials and their manufacture; and with knowledge will come enthusiasm—enthusiasm which will stand us in excellent stead at the bedside of the patient. All this is as it should be. Unless we begin at the beginning we find ourselves lost in fogs as we get into higher regions. Materia medica, like anatomy and physiology, must be studied thoroughly, especially as to its elements, or successful practitioners will be few and far between.

There is an admirable terseness and brevity about the style of the book. The slipshod methods so common, and so altogether abominable, in medical literature are conspicuous by their absence. Dr. Hale White respects the English language, and himself and his readers at the same time. An astonishing saving of time and temper are thus effected for the reader. He reads with pleasure, and from the beginning to the end of a chapter is never once tempted to swear. Here, for example, is a specimen of Dr. White's style. The paragraph is headed "Dispensing the Prescription," and runs thus: "The dispenser should bear the following rules in mind: (1) Read the prescription through first. (2) Next write the directions, so that they have time to dry. (3) Solution by heat should not be used if more of the salt is ordered than will dissolve in cold water. In such a case it must be suspended. (4) With fluids, measure them in such an order that the measure-glass shall be finally rinsed out with the vehicle. (5) Use glass scale pans. (6) Clean and put away everything directly after use. (7) If in the slightest doubt, ask the prescriber. (8) If finally the prescription contains any insoluble matter, label 'Shake the bottle.' (9) If the medicine is very poisonous, label it as such, and use a distinctive bottle. (10) If for outward application only, say so." This is excellent as an elementary lesson in dispensing.

But it is mainly the prescriber whom Dr. Hale White has had in view, and for his guidance there are well-planned, well-reasoned, and philosophic chapters of very great value indeed. The human body is mapped out into parts and systems, and the physiological actions of drugs are clearly set forth in their relations to those parts and systems. A careful attempt is made to expound all that has been experimentally ascertained of the actions of drugs, as well as to give the accumulated results of clinical experience. So far as is possible the information given is scientific and precise. A few headings of divisions will indicate how thoroughly and systematically all this is done. For example, we have briefly expressed divisions on "drugs acting on the blood," "drugs acting on the cardiac mechanism," "drugs acting on the vessels," "drugs acting on the skin," "drugs acting on the bodily heat," "drugs acting on respiration," "drugs acting on the digestive apparatus," and so on. Not only are these great systems therapeutically considered in relation to the drugs to be employed upon them, but every part of which they are constituted, with the drugs which act upon it has been studied, and is dealt with in brief and careful detail. In short, Dr. Hale White has offered to the profession a thoroughly systematic and scientific work on materia medica containing in intelligible form all the best knowledge of the subject, both old and new.

The student is always an important person to be considered in the preparation of works on materia medica.

* "Materia Medica, Pharmacy, Pharmacology, and Therapeutics." By W. Hale White, M.D., F.R.C.P. (London: J. and A. Churchill.)

For the real student it would be difficult to imagine a work more suitable than Dr. Hale White's. The men who read for honours and for the higher examinations will find it invaluable, since it is at once systematic and thorough, full and brief. The ordinary student will not, however, be disappointed. The common knowledge required at all the examining boards is made available in a systematic and easily remembered form. Indeed, we shall be very much surprised if, in the course of a year or two, Dr. Hale White's admirable book does not take the premier place among all the works of its class.

